# Global update on the susceptibility of human influenza viruses to neuraminidase inhibitors, 2013–2014

**DOI:** 10.1016/j.antiviral.2015.02.003

**Published:** 2015-02-23

**Authors:** Emi Takashita, Adam Meijer, Angie Lackenby, Larisa Gubareva, Helena Rebelo-de-Andrade, Terry Besselaar, Alicia Fry, Vicky Gregory, Sook-Kwan Leang, Weijuan Huang, Janice Lo, Dmitriy Pereyaslov, Marilda M. Siqueira, Dayan Wang, Gannon C. Mak, Wenqing Zhang, Rod S. Daniels, Aeron C. Hurt, Masato Tashiro

**Affiliations:** a World Health Organization Collaborating Centre for Reference and Research on Influenza, National Institute of Infectious Diseases, Gakuen 4-7-1, Musashimurayama, Tokyo 208-0011, Japan; b National Institute for Public Health and the Environment, PO Box 1, 3720 BA Bilthoven, The Netherlands; c Public Health England Colindale, 61 Colindale Avenue, London NW9 5EQ, United Kingdom; d World Health Organization Collaborating Centre for the Surveillance, Epidemiology and Control of Influenza, Centers for Disease Control and Prevention, 1600 Clifton RD NE, MS-G16 Atlanta, GA, United States; e Instituto Nacional de Saúde, Av. Padre Cruz, 1649-016 Lisboa, Portugal; f Faculdade de Farmácia, Universidade de Lisboa, Av. Prof. Gama Pinto, 1649-003 Lisboa, Portugal; g Global Influenza Programme, World Health Organization, Avenue Appia 20, 1211 Geneva 27, Switzerland; h World Health Organization Collaborating Centre for Reference and Research on Influenza, MRC-National Institute for Medical Research, The Ridgeway, Mill Hill, London NW7 1AA, United Kingdom; i World Health Organization Collaborating Centre for Reference and Research on Influenza, VIDRL, At the Peter Doherty Institute for Infection and Immunity, Melbourne, VIC 3000, Australia; j World Health Organization Collaborating Centre for Reference and Research on Influenza, Chinese National Influenza Center, National Institute for Viral Disease Control and Prevention, Chinese Center for Disease Control and Prevention, 155 Changbai Road, Changping District, Beijing 102206, China; k Public Health Laboratory Centre, 382 Nam Cheong Street, Shek Kip Mei, Kowloon, Hong Kong, China; l Division of Communicable Diseases, Health Security, & Environment, World Health Organization Regional Office for Europe, UN City, Marmorvej 51, DK-2100 Copenhagen ø, Denmark; m Respiratory Viruses Laboratory/IOC, FIOCRUZ, Av Brasil, 4365 Rio de Janeiro, Brazil; n University of Melbourne, Melbourne School of Population and Global Health, Melbourne, VIC 3010, Australia

**Keywords:** Influenza virus, Antiviral resistance, Neuraminidase inhibitors, Oseltamivir, Global analysis, Reduced susceptibility

## Abstract

Four World Health Organization (WHO) Collaborating Centres for Reference and Research on Influenza and one WHO Collaborating Centre for the Surveillance, Epidemiology and Control of Influenza (WHO CCs) tested 10,641 viruses collected by WHO-recognized National Influenza Centres between May 2013 and May 2014 to determine 50% inhibitory concentration (IC_50_) data for neuraminidase inhibitors (NAIs) oseltamivir, zanamivir, peramivir and laninamivir. In addition, neuraminidase (NA) sequence data, available from the WHO CCs and from sequence databases (*n* = 3206), were screened for amino acid substitutions associated with reduced NAI susceptibility. Ninety-five per cent of the viruses tested by the WHO CCs were from three WHO regions: Western Pacific, the Americas and Europe. Approximately 2% (*n* = 172) showed highly reduced inhibition (HRI) against at least one of the four NAIs, commonly oseltamivir, while 0.3% (*n* = 32) showed reduced inhibition (RI). Those showing HRI were A(H1N1)pdm09 with NA H275Y (*n* = 169), A(H3N2) with NA E119V (*n* = 1), B/Victoria-lineage with NA E117G (*n* = 1) and B/Yamagata-lineage with NA H273Y (*n* = 1); amino acid position numbering is A subtype and B type specific. Although approximately 98% of circulating viruses tested during the 2013–2014 period were sensitive to all four NAIs, a large community cluster of A(H1N1)pdm09 viruses with the NA H275Y substitution from patients with no previous exposure to antivirals was detected in Hokkaido, Japan. Significant numbers of A(H1N1)pdm09 NA H275Y viruses were also detected in China and the United States: phylogenetic analyses showed that the Chinese viruses were similar to those from Japan, while the United States viruses clustered separately from those of the Hokkaido outbreak, indicative of multiple resistance-emergence events. Consequently, global surveillance of influenza antiviral susceptibility should be continued from a public health perspective.

## 1. Introduction

Neuraminidase inhibitors (NAIs) are currently the only licenced antiviral drugs which are effective for the treatment or prophylaxis of seasonal influenza. National and regional antiviral stockpile policies, where they exist, rely primarily on oseltamivir and to a far lesser extent zanamivir, both of which have been approved for use in many countries since 1999–2000. In Japan, two other NAIs, peramivir and laninamivir, have been approved for seasonal use, and favipiravir (T705; Toyama Chemicals), a viral RNA dependent RNA polymerase inhibitor, has recently been approved for pandemic preparedness stockpiling only (http://www.toyama-chemical.co.jp/eng/news/news140324e.html). Peramivir is also approved for use in the Republic of Korea, China, and the United States.

Experience from 2007–2008, when the former seasonal A(H1N1) virus acquired oseltamivir resistance due to an H275Y neuraminidase (NA) amino acid substitution and spread globally within 12 months, has demonstrated that surveillance for NAI-resistant viruses is essential both to guide seasonal clinical management and inform pandemic preparedness strategies (Lackenby et al., 2008; Collins et al., 2009; Dharan et al., 2009; García et al., 2009; Hauge et al., 2009; Hurt et al., 2009; Meijer et al., 2009; Ujike et al., 2010).

The former seasonal A(H1N1) H275Y virus exhibited highly reduced inhibition (HRI) by oseltamivir and peramivir *in vitro* and was shown to be clinically resistant to oseltamivir (Kawai et al., 2009; Dharan et al., 2010; Matsuzaki et al., 2010; Saito et al., 2010). Additional NA substitutions (R222Q, V234M, D344N and D354G) compensated for the detrimental effect of the H275Y substitution on virus fitness, allowing the virus to spread efficiently (Bloom et al., 2010; Rameix-Welti et al., 2011; Abed et al., 2011; Bouvier et al., 2012).

The World Health Organization (WHO) Global Influenza Surveillance and Response System (GISRS) expert working group on surveillance of influenza antiviral susceptibility (WHO-AVWG) was established in 2011 to provide advice on GISRS surveillance strategies for influenza antiviral susceptibility and to provide practical guidance to WHO-recognized National Influenza Centres (NICs) (WHO, 2012, 2013).

To standardise interpretation and reporting of NAI susceptibility of influenza viruses to individual NAIs, clear definitions were formulated by the WHO-AVWG using 50% inhibitory concentration (IC_50_; the concentration of drug required to inhibit a standardised amount of NA activity by 50%) fold-change thresholds, compared to the median for viruses from the same type/subtype/lineage showing ‘normal inhibition’ (NI) (WHO, 2012). Those showing ‘reduced inhibition’ (RI) are influenza A viruses that have a 10- to 100-fold increase in IC_50_, or influenza B viruses with a 5- to 50-fold increase in IC_50_. Viruses showing HRI are influenza A viruses with > 100-fold increase in IC_50_ or influenza B viruses with >50-fold increase in IC_50_ (WHO, 2012).

Recently, we published a global update on the antiviral susceptibility of human influenza viruses collected by NICs between May 2012 and May 2013 as the first of a series of annual reports (Meijer et al., 2014). Only 0.2% (*n* = 27) of 11,387 viruses tested showed HRI against at least one of the four NAIs, usually oseltamivir, and mainly in A(H1N1)pdm09 viruses (21/27). Despite >99% of circulating viruses being sensitive to all four NAIs during the 2012–2013 period, localised community circulation of influenza viruses with RI or HRI has occurred in recent years, most notably with A(H1N1)pdm09 viruses containing the H275Y NA substitution (Hurt et al., 2012; Garg et al., 2013). Animal models have shown that A(H1N1)pdm09 H275Y viruses with additional NA amino acid substitutions, V241I and N369K, have increased replication and transmission fitness (Butler et al., 2014; Abed et al., 2014). Importantly, >97% of the N1 sequences from circulating A(H1N1)pdm09 viruses in 2012–2013 contained the two NA substitutions V241I and N369 K that improve viral fitness of the variant virus (Meijer et al., 2014). These observations, together with those from the former seasonal A(H1N1) 2007–2008 event, illustrate the potential for the global emergence of fit A(H1N1)pdm09 viruses with HRI by oseltamivir and peramivir.

Here, we analysed the NAI susceptibility data for influenza viruses collected across 113 countries by GISRS laboratories between May 2013 and May 2014 (subsequently referred to as 2013–2014).

## 2. Overall analysis of phenotypic antiviral susceptibility data from WHO CCs

NIC within each country receives or collects clinical specimens from the national laboratory network in order to conduct preliminary analyses. NICs also send representative virus isolates to at least one of the five WHO CCs (Atlanta, United States; Beijing, China; London, United Kingdom; Melbourne, Australia, and Tokyo, Japan; http://www.who.int/influenza/gisrs_laboratory/collaborating_centres/list/en/) for more advanced analyses. At the WHO CCs, viruses are in general passaged one or two times in MDCK cells before being subjected to phenotypic antiviral susceptibility testing.

Five WHO CCs provided IC_50_ and NA amino acid substitution data for virus isolates, notably for those showing RI or HRI by NAIs, recovered from clinical specimens collected between week 21/2013 (20/5/2013) through week 20/2014 (18/5/2014). When available, patient-specific epidemiologic data such as gender, age, geographic location, healthcare setting (community, hospitalised and sentinel/non-sentinel specimen collection), antiviral treatment history and immune status, were included in the analyses. All five WHO CCs tested for oseltamivir and zanamivir susceptibility, and additionally the Atlanta, Melbourne and Tokyo WHO CCs tested for peramivir and laninamivir susceptibility (Supplementary Table 1).

**Fig. 1.**
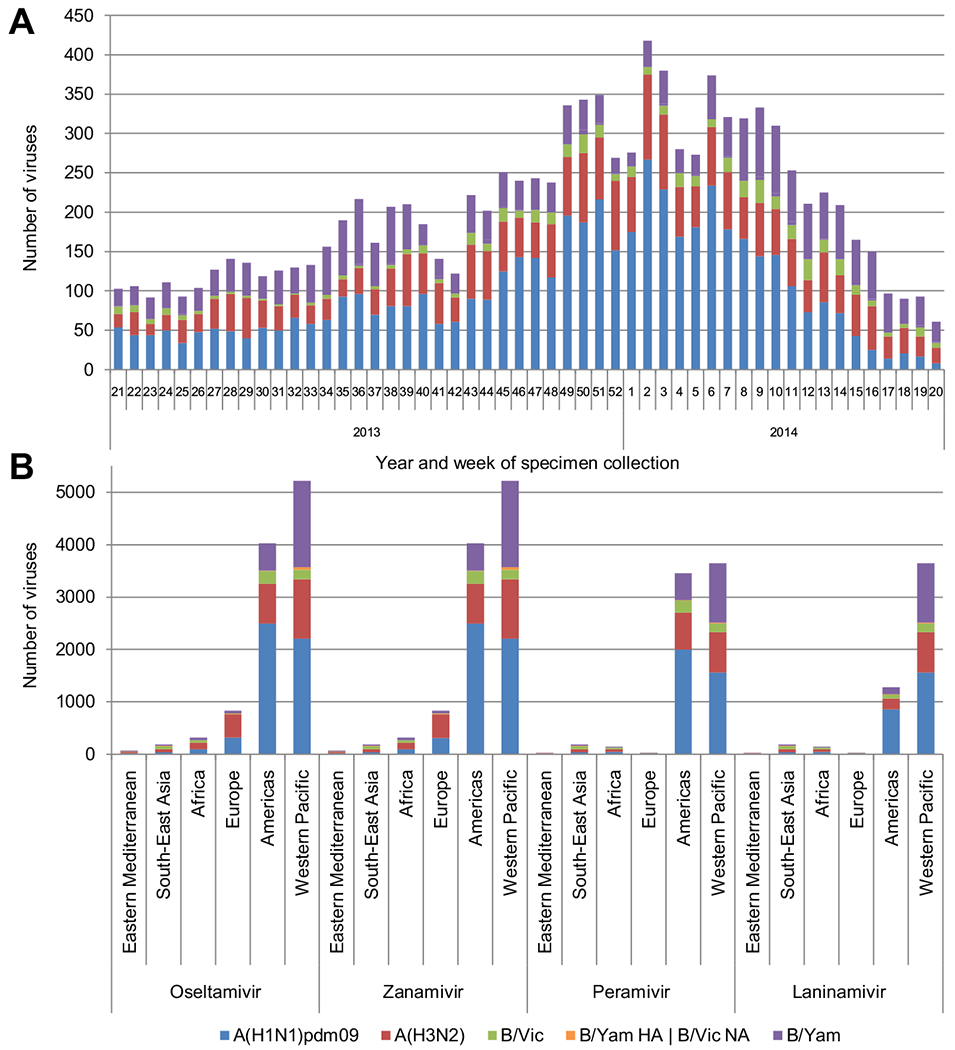
Influenza viruses collected and tested for neuraminidase inhibitors (NAI) susceptibility during 2013–2014. (A) Week of specimen collection and virus type/subtype/lineage; for specimens tested, a small peak in specimen collection during the Southern Hemisphere winter and a prominent peak during the Northern Hemisphere winter were observed. (B) Number of viruses tested for susceptibility to the four NAIs by WHO region. B/Yamagata-lineage haemagglutinin – B/Victoria-lineage neuraminidase reassortants are shown separately. The greatest numbers of viruses tested were from the Western Pacific Region and the Americas. Almost all viruses were tested for susceptibility to oseltamivir and zanamivir and a high proportion against peramivir and laninamivir.

The WHO CCs tested 10,641 viruses from the 2013–2014 period for NAI susceptibility using local adaptations of the fluorescence-based NA enzyme inhibition assay described by Potier et al. (1979) (Supplementary Table 1). The majority of viruses tested were derived from community surveillance specimens, typically collected for influenza diagnosis, and therefore prior to any NAI treatment. However, while antiviral treatment information was not available for many of the specimens, a proportion of viruses were probably derived from patients during or after treatment with NAI, in hospital or community settings. The number of viruses tested was well distributed across the time period but with a small peak during the Southern Hemisphere winter and a prominent peak during the Northern Hemisphere winter (Fig. 1A).

During the 2013–2014 period, B/Yamagata-lineage haemagglutinin (HA) – B/Victoria-lineage NA reassortants were detected worldwide, notably in China. While not all B viruses had HA and NA genes sequenced, to assess their reassortant status, 65 known reassortants were tested and results allocated to the B/Victoria-lineage dataset. Over the 12 months 5152 (48%) A(H1N1)pdm09, 2574 (24%) A(H3N2), 2311 (22%) B/Yamagata-lineage and 604 (6%) B/Victoria-lineage viruses were tested.

By WHO region (http://www.who.int/about/structure/en/), 49% of viruses tested originated from the Western Pacific Region, 38% from the Americas, 8% from Europe, <3% from Africa, <2% from South-East Asia and <1% from the Eastern Mediterranean Region (Fig. 1B).

**Fig. 2.**
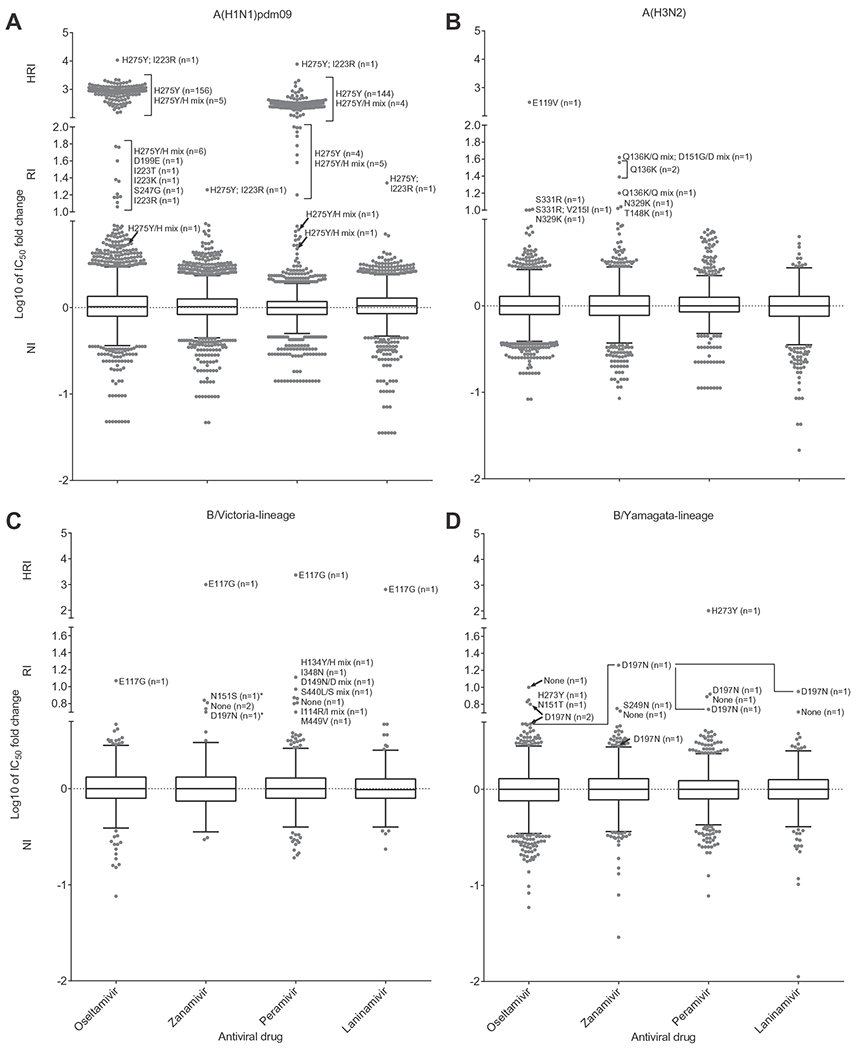
Column-scatter plots of log-transformed IC_50_ fold-change values. Data are presented by virus subtype or lineage (A, A(H1N1)pdm09; B, A(H3N2); C, B/Victoria-lineage; D, B/Yamagata-lineage) and neuraminidase inhibitor (NAI) (labelled on the X-axis: oseltamivir, zanamivir, peramivir, laninamivir). Panel C also contains B/Yamagata-lineage haemagglutinin – B/Victoria-lineage neuraminidase (NA) reassortants, of which those with amino acid substitutions are indicated with an asterix (*) in the zanamivir column. The boxes indicate the 25–75 percentile and the whiskers stretch to the lowest and highest value within 1.5 times the interquartile region value from both the 25 and 75 percentile values respectively (Tukey’s definition). The Y-axes have been split into 3 compartments according to the WHO-AVWG recommended thresholds for normal inhibition (NI) (A viruses <10-fold; B viruses <5-fold), reduced inhibition (RI) (A viruses 10- to 100-fold; B viruses 5- to 50-fold), and highly reduced inhibition (HRI) (A viruses >100-fold; B viruses >50-fold). For RI and HRI viruses that have been sequenced the determined amino acid substitutions are shown; amino acid position numbering is A subtype and B type specific. Connecting lines for one virus with NA D197N indicate the differences in IC_50_ fold-changes with the four NAIs.

Due to differences in phenotypic NA inhibition assay methodology between the WHO CCs (Supplementary Table 1) all raw IC_50_ data were converted into relative fold-change values to facilitate pooled analysis of the data from all five WHO CCs (Meijer et al., 2014). Box-and-whisker plots based on log-transformed IC_50_ fold-change data were generated using Tukey’s method to display the range of data and outliers (Fig. 2). Additionally, the box-and-whisker plots were constructed with the Y-axis split into three sections indicating the IC_50_ fold-change range for viruses classified as NI, RI or HRI based on the criteria above (Fig. 2). Among 10,641 viruses tested, 204 viruses (2%) showed RI or HRI to one or more of the NAIs (Fig. 2 and Table 1). The NA genes of all 204 RI/HRI viruses were sequenced, together with those present in clinical specimens from which 67 of the viruses were recovered. Compared to the consensus NA sequences of wild-type viruses showing NI, 198 of the RI/HRI viruses encoded an amino acid substitution in the NA glycoprotein, of the clinical specimens available (*n* = 67), the same mutations to those observed in the isolate were also present in 63 corresponding clinical specimens (Table 1).

## 3. A(H1N1)pdm09 viruses showing RI or HRI

**Fig. 3.**
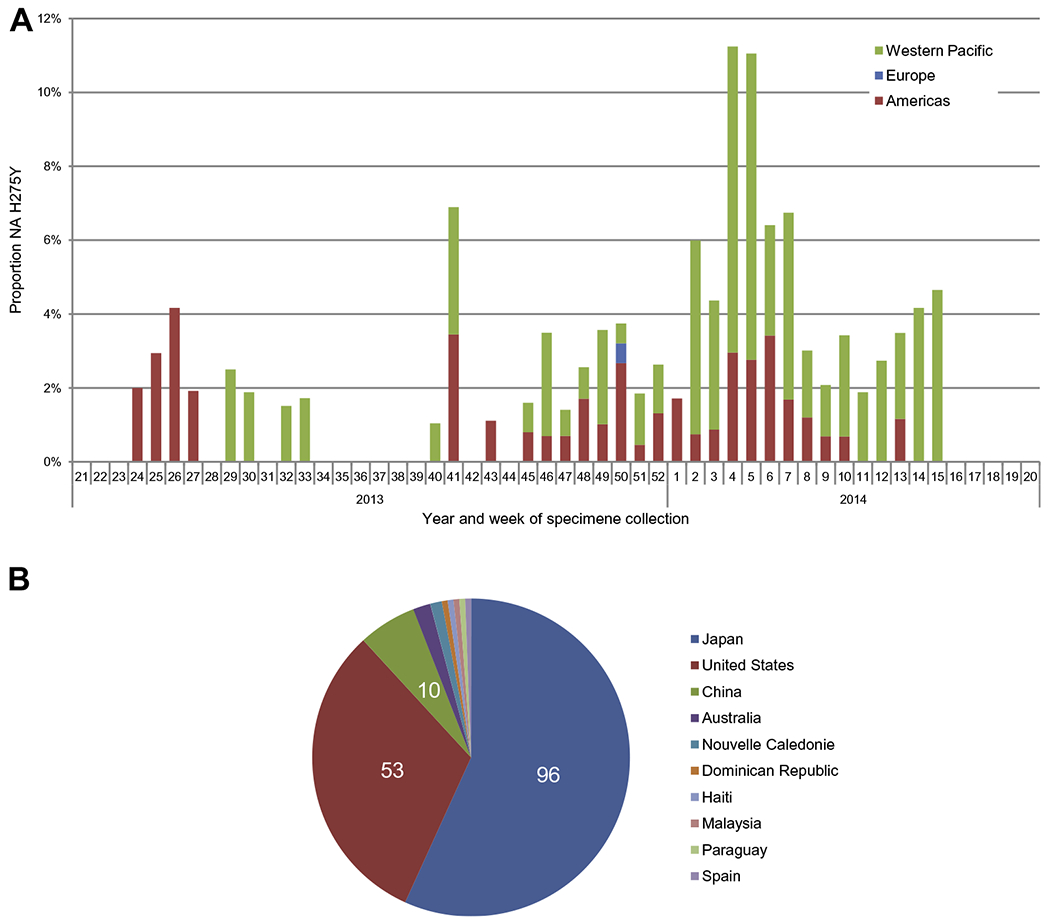
Specimen collection timing and geographic distribution of 169 neuraminidase (NA) H275Y containing A(H1N1)pdm09 viruses. (A) NA H275Y containing A(H1N1)pdm09 viruses by year and week and WHO region. Proportions of the total 5152 A(H1N1)pdm09 viruses tested phenotypic at the WHO CCs by week. (B) Distribution of NA H275Y containing A(H1N1)pdm09 viruses tested phenotypic and genotypic by country.

Of 5152 A(H1N1)pdm09 viruses tested, 175 (3%) showed RI or HRI by one or more of the NAIs (Fig. 2 and Table 1), of which the NA amino acid substitution H275Y was present in 169 of those. Twelve of the 169 H275Y variants harboured a mixed population of H275Y variant virus and H275 wild type virus. One H275Y variant with an additional NA I223R substitution was detected in Japan from a hospitalised patient treated with peramivir; this dual substitution has been reported previously by the United States, in a child after prolonged treatment with oseltamivir (Nguyen et al., 2010). The timing of specimen collection and geographic distribution of the 169 H275Y variant viruses are shown in Fig. 3. The H275Y variants were detected between weeks 24/2013 and 15/2014 (Fig. 3A), with 112 (66%) detected in the Western Pacific region, 56 (33%) in the Americas and one (1%) in Europe, from 10 countries in total (Fig. 3B). The NA H275Y substitution conferred 151- to 2212-fold higher oseltamivir IC_50_ values and 87- to 2045-fold higher peramivir IC_50_ values compared to wild type viruses, but had little or no effect on zanamivir or laninamivir susceptibility (Table 1). The H275Y/H mixed variants showed 5.4- to 584-fold higher oseltamivir IC_50_ values and 4.8- to 599-fold higher peramivir IC_50_ values, depending on the proportion of the H275Y variant in the mixed population. The H275Y/I223R variant showed HRI with very high increases in oseltamivir (10,739-fold) and peramivir (7709-fold) IC_50_ values and RI by zanamivir and laninamivir, with 18.1- and 22-fold IC_50_ increases respectively, compared to values for A(H1N1)pdm09 viruses displaying NI by the four NAIs. The NA H275Y substitution was confirmed in 58 clinical specimens for which sequence results were available and the NA I223R substitution was confirmed in the clinical specimen from which the H275Y/I223R variant virus was isolated (Table 1).

Of the H275Y variant viruses detected in 2013–2014 where patient setting information was available, 82% were from non-hospitalised patients (Table 1). In addition, only two of the 99 patients for whom immune status information was available, were reported as being immunocompromised. Based on available antiviral treatment information, 10 of the 12 H275Y/H mixed variants were recovered from patients during or after treatment with oseltamivir or peramivir. Of the remaining H275Y variants recovered, 82% were from patients had not been treated with NAIs before specimen collection, suggesting that there is potential for spontaneous emergence and spread of these resistant viruses in the community. Indeed, a cluster of A(H1N1)pdm09 viruses with the NA H275Y substitution was detected in 39 (29%) of 135 untreated community cases in Sapporo, capital of Hokkaido, Japan (described in detail in Takashita et al., 2014). In the United States, 57 oseltamivir-resistant A(H1N1)pdm09 viruses were detected in 20 states with a majority being collected from patients not exposed to oseltamivir (Okomo-Adhiambo et al., 2015). In China, nine H275Y variant viruses were detected in nine provinces, while the oseltamivir exposure history of four patients was unknown, the remaining five had no exposure to oseltamivir.

Other NA amino acid substitutions (D199E, I223K, I223T, I223R and S247G) were detected in viruses with elevated IC_50_ values (Fig. 2 and Table 1). The clinical specimens yielding four of these isolates were available and all contained the corresponding substitutions; no matching clinical specimen was available for the viruses carrying NA D199E or NA I223R substitutions. The IC_50_ fold-changes for virus with the dual substitution NA H275Y/I223R compared to those of viruses with NA H275Y or NA I223R only (Fig. 2), shows clearly the synergistic effect of this dual substitution and induction of RI or HRI for all four NAIs tested (Tan et al., 2013).

## 4. A(H3N2) viruses showing RI or HRI

Nine (0.3%) A(H3N2) viruses out of 2574 tested showed RI or HRI by oseltamivir or zanamivir (Fig. 2 and Table 1). Three of these had a NA Q136K amino acid substitution (Table 1). Available clinical specimens yielding two of these isolates did not contain the mutation conferring the amino acid substitution prior to cell culture. However, NA Q136K substitution has been reported previously in clinical specimens (Dapat et al., 2010). One A(H3N2) virus from the United States contained a NA E119V substitution which conferred HRI by oseltamivir but had no effect on susceptibility to the other NAIs. The E119V variant virus came from an oseltamivir-treated patient. The NA E119V substitution was detected in the corresponding clinical specimen. NA amino acid substitutions T148K, N329K, S331R and V215I were detected in viruses with IC_50_ fold-change values that were close to the intersect between NI and RI categories for oseltamivir and/or zanamivir. NA T148K and D151G substitutions can emerge during culturing of A(H3N2) viruses (Tamura et al., 2013).

## 5. B/Victoria-lineage viruses showing RI or HRI

Of 604 B/Victoria-lineage viruses tested, 12 (2%) showed RI or HRI by one or more of the NAIs (Fig. 2 and Table 1). One B/Victoria-lineage virus from Bangladesh with a NA E117G substitution showed HRI by three of the four NAIs tested with a 2326-fold increase in peramivir IC_50_, a 1010-fold increase in zanamivir IC_50_, a 649-fold increase in laninamivir IC_50_ and RI with a 12-fold increase in oseltamivir IC_50_ compared to values for B/Victoria-lineage viruses displaying NI by the four NAIs (Table 1). NA amino acid substitutions N151S and D197N were detected in B/Yamagata-lineage HA – B/Victoria-lineage NA reassortants. All other substitutions identified in the isolates that were classified as having RI to zanamivir or peramivir require further analysis to assess whether the amino acid substitutions observed are responsible for the changes in IC_50_. Three B/Victoria-lineage viruses had IC_50_ values that were close to the intersect between NI and RI categories for zanamivir or peramivir, but no NA amino acid substitutions were detected on sequencing.

## 6. B/Yamagata-lineage viruses showing RI or HRI

Eight (0.3%) B/Yamagata-lineage viruses out of 2311 tested showed RI or HRI by one or more of the NAIs (Fig. 2 and Table 1). One B/Yamagata-lineage virus from Macau, China contained an NA H273Y substitution which conferred HRI (103-fold increase) by peramivir and RI (7.1-fold increase) by oseltamivir but had no effect on susceptibility to zanamivir and laninamivir (Table 1). The two viruses carrying NA D197N substitution yielded different patterns of NI and RI by all NAIs, around the intersect between NI and RI categories; only for peramivir did both show RI. The NA D197N substitution has been observed before in B/Yamagata-lineage virus and shown to confer RI by zanamivir and peramivir (Oakley et al., 2010). One of the two D197N variant viruses, came from a patient during treatment with zanamivir, but no information was available for the second patient. NA amino acid substitutions N151T and S249N were detected in viruses with elevated IC_50_ values (Table 1). The clinical specimen yielding the S249N variant also contained the substitution. The clinical specimen yielding the N151T variant did not contain the substitution. Further investigation is needed to assess the role of these substitutions in altering NAI susceptibility. Three B/Yamagata-lineage viruses had IC_50_ values that were around the intersect between NI and RI categories, but NA-gene sequencing revealed no NA amino acid substitutions.

## 7. Frequency of RI and HRI conferring NA amino acid substitutions in sequence databases

**Fig. 4.**
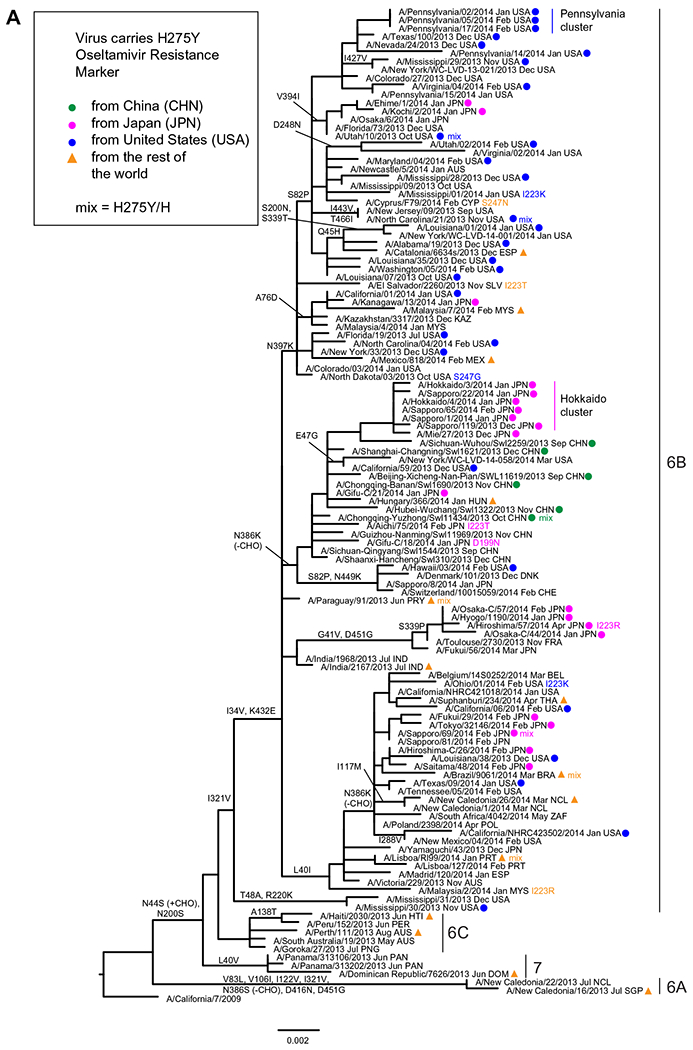

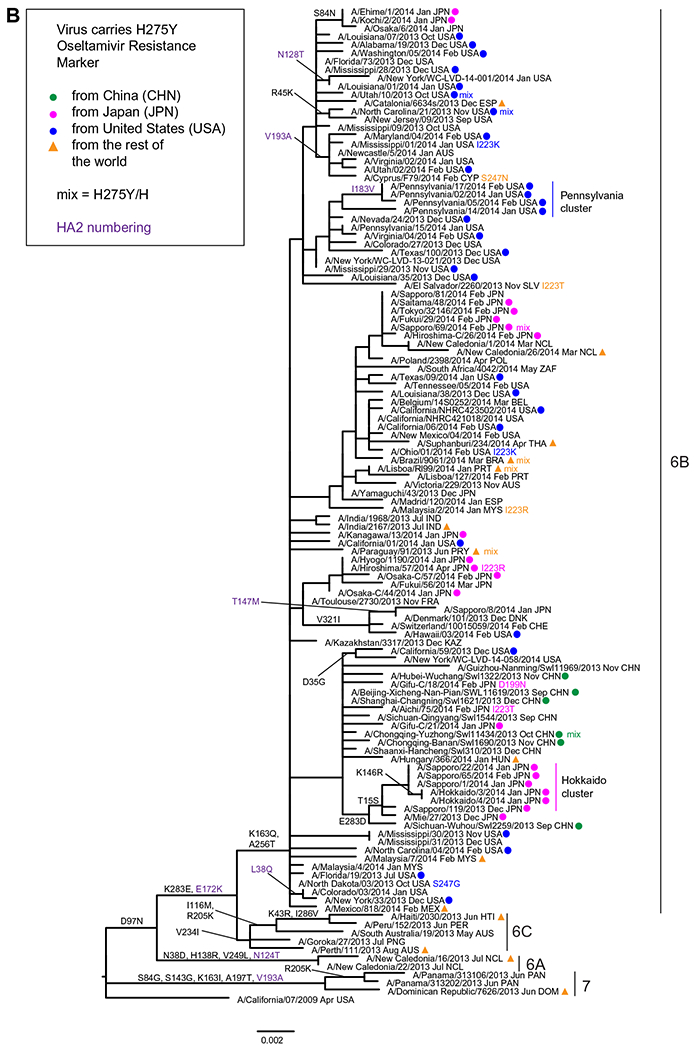
Evolutionary relationships among influenza A(H1N1)pdm09 virus neuraminidase (NA) and haemagglutinin (HA) genes. The phylogenetic trees were constructed using RAxML (http://sco.h-its.org/exelixis/software.html), drawn using FigTree (http://tree.bio.ed.ac.uk/software/figtree/) and annotated using Adobe Illustrator (http://www.adobe.com/products/illustrator.html). NA (A) and HA (B) gene sequences of 119 representative A(H1N1)pdm09 viruses, with collection dates in the timeframe 20 May 2013–18 May 2014 were analysed: the virus selection included 50 with NA H275 and 62 with NA H275Y amino acid substitution and seven with NA H275Y/H (mix). A/California/07/2009 virus was used as a reference for ancestry (root) and numbering. Both trees are annotated in the same way: (i) viruses carrying NA H275Y substitution are marked by country of origin (China, Japan, United States) using coloured circles (see key), with triangles representing the rest of the world; (ii) viruses carrying other/additional NA amino acid substitutions at positions known to be implicated in reduced susceptibility to at least one neuraminidase inhibitor are indicated in the appropriate colour at the end of virus names (positions assessed were 119 (0), 199 (1), 223 (6), 247 (2), and 295 (0) with the number of viruses carrying such substitutions indicated in parentheses); (iii) clusters of viruses representing outbreaks of oseltamivir resistant A(H1N1)pdm09 viruses in Hokkaido (Japan) and Pennsylvania (United States) are indicated; (iv) bars indicate the proportion of nucleotide changes between sequences. On the NA tree (A) amino acid substitutions associated with loss (−CHO) or gain (+CHO) of potential N-linked glycosylation sites are shown. On the HA tree (B) amino acid substitutions in HA2 are shown in purple.

We screened NA sequences from viruses collected during the 2013–2014 period that had been deposited in the Global Initiative on Sharing All Influenza Data (GISAID) database, at www.gisaid.org, and the National Center for Biotechnology Information Influenza Virus Resource (NCBI-IVR), at www.ncbi.nlm.nih.gov/genomes/FLU/FLU.html, for amino acid substitutions which are known to confer clinical resistance (e.g. H275Y in A(H1N1)pdm09 viruses), or are known to occur clinically. A list of the amino acid substitutions that are relevant for clinical and surveillance interpretation, reviewed and updated in May 2014 by the WHO-AVWG, is available at: http://www.who.int/influenza/gisrs_laboratory/antiviral_susceptibility/nai_overview/en/ (accessed 21 November 2014). A total of 3206 sequences from GISAID and NCBI-IVR databases were analysed for the presence of key NA amino acid substitutions, following curation for quality, sequence length and duplication (Supplementary Table 2). The majority of sequences in the databases were from viruses collected through the GISRS network and characterised via phenotypic analysis in GISRS laboratories. Of 1328 N1 sequences from A(H1N1)pdm09 viruses, 165 (12%) contained NA H275Y substitution, of which 16 showed NA 275Y/H polymorphism and one carried NA H275Y/I223R dual substitutions (Table 2). The H275Y/I223R variant virus had been analysed by NA inhibition assay at a WHO CC and is included in Fig. 2 and Table 1. The 165 H275Y variant viruses were derived from 11 countries. Seven H275Y variant viruses were in the sequence database but not present in the WHO CCs IC_50_ dataset. Phylogenetic analysis of NA (Fig. 4A) and HA (Fig. 4B) gene sequences showed, in both phylogenetic trees, that the H275Y variant viruses did not emerge from a common source. However, the H275Y variants from China were genetically similar to those from the Hokkaido cluster.

Analysis of the available sequences also identified 25 N1 sequences from A(H1N1)pdm09 viruses containing D199N substitution, of which seven originated from Bulgaria, and four influenza B NA sequences containing D197N substitution (Table 3). Twentyone of the 25 N1 D199N variants and three of the four B NA D197N variants had been analysed by NA inhibition assay at the WHO CCs. The A(H1N1)pdm09 viruses with NA D199N substitution showed NI. The influenza B viruses containing NA D197N substitution showed RI to one or more of the NAIs. These results are consistent with the AVWG table (http://www.who.int/influenza/gisrs_laboratory/antiviral_susceptibility/nai_overview/en/). Two N2 sequences contained E119V substitution, with no evidence for 119E/V mixtures, and both E119V variants were analysed by NA inhibition assay at the WHO CCs. One showed HRI to oseltamivir and is included in Fig. 2 and Table 1 and the other showed NI. In the AVWG table, it is listed that the NA E119V substitution confers 18-fold (RI) to 2057-fold (HRI) increases in oseltamivir IC_50_.

## 8. Concluding remarks

The WHO-AVWG was able to perform this global analysis on influenza antiviral susceptibility thanks to NICs within the WHO GISRS fulfilling their Terms of Reference by collecting influenza virus positive clinical specimens and sharing a representative proportion of them, or viruses recovered, with the WHO CCs for further detailed characterisation (Kitler et al., 2002).

Based on our current analysis, approximately 98% of all viruses circulating during 2013–2014 were sensitive to all four NAIs and therefore these drugs remain an appropriate choice for the treatment and prophylaxis of influenza virus infections. However, a large community cluster of A(H1N1)pdm09 viruses with the NA H275Y substitution occurred in Hokkaido, Japan between November 2013 and February 2014. In 2011, a widespread community cluster of NA H275Y variant A(H1N1)pdm09 viruses occurred in Newcastle, Australia (Hurt et al., 2012). The latter H275Y variant viruses possessed V241I and N369K substitutions in the NA which partially overcame the detrimental effects of the H275Y substitution on virus fitness (Butler et al., 2014; Abed et al., 2014). Almost all recently circulating A(H1N1)pdm09 viruses possess the NA V241I and N369K substitutions, indicating increased risk of H275Y variant viruses emerging and spreading globally. The Hokkaido cluster viruses carried these two substitutions and shared NA N386K substitution with the H275Y variant viruses detected in China. Therefore, the H275Y variant viruses of the Hokkaido cluster and those of China may be derived from a common ancestor.

Despite low numbers of virus isolates from Africa, South East Asia and East Mediterranean regions being available for analysis, this pooled analysis of IC_50_ data from the five WHO CCs offers the best opportunity to gain a robust global picture of the incidence of RI/HRI by NAIs and the relatedness of the NAI resistant viruses.

The majority of NA sequences in the GISAID sequence database are deposited by the WHO CCs. There are an increasing number of GISRS NICs with NA sequencing capability, but this is not reflected in the current analysis as many of the submitted sequences are incomplete and do not cover all known reduced NAI susceptibility conferring NA amino acid substitution positions. These laboratories should be encouraged to perform full-length NA-gene sequencing and submit available sequences in a timely manner as it would add significant value to global NAI susceptibility surveillance efforts.

The issues of incomplete clinical and epidemiologic data accompanying clinical specimens or virus isolates being referred to the WHO CCs remains a limitation in determining the significance of RI/HRI virus detection. NICs often cannot obtain this information on all samples, particularly when they are received from non-sentinel surveillance systems. Follow-up of individual samples requires significant, often one-to-one interaction with sub-national laboratories or physicians which is not possible for some NICs. In addition, NAI susceptibility analysis of samples for surveillance purposes may not be performed in a timely manner, making the collection of clinical and epidemiologic data for characterisation of the risk factors for NAI-RI/HRI virus infection difficult. Knowledge of whether the sample originated from patients in the community or hospitals can more easily be obtained. The WHO-AVWG is currently developing the WHO database (FluNet) to collect this basic information, together with H275Y screening data for influenza A(H1N1)pdm09 viruses, from all NICs who perform such testing. This basic information, which can also be supplied with the samples referred to the WHO CCs, does at least facilitate screening for increased incidence of viruses with HRI from untreated patients in the community, such as detected in Japan in 2013–2014 and Australia in 2011.

This is the second global update on influenza NAI susceptibility based on analysing data generated by WHO CCs on samples received from the GISRS laboratories. Overall, the proportion of viruses showing RI/HRI was similar between the two years, approximately 1% in 2012–2013 and 2% in 2013–2014. The slight increase in 2013–2014 is due to several clusters of untreated cases of A(H1N1)pdm09 viruses carrying NA H275Y substitution in China, Japan and the United States. Only 1% of A(H1N1)pdm09 viruses exhibited RI/HRI in 2012–2013, compared with 3% in 2013–2014. Rates of RI/HRI detection in A(H3N2) (0.4% vs 0.3%), B/Victoria- (1% vs 2%) and B/Yamagata- (0.3% both years) lineage viruses have remained very similar for the two seasons analysed to date. Prevalence of RI/HRI viruses has consistently been higher for B/Victoria-lineage over B/Yamagata-lineage viruses. This could be a reflection of the overall number of viruses analysed (more B/Yamagata-lineage in both seasons) but could also be an indication of a slightly higher tendency for B/Victoria-lineage NA to tolerate RI/HRI conferring substitutions. This could merit further investigation, taking into account reassortant events between the two lineages, notably the recent emergence of B/Yamagata-lineage HA – B/Victoria-lineage NA reassortants.

## Supplementary Material

mmc1

mmc2

mmc3

## Figures and Tables

**Table 1:** Virus and patient characteristics of 204 viruses showing RI or HRI, tested by WHO CCs.^a^

Virus	*n*	IC_50_ fold-change compared to reference median IC_50_ values^b^				NA-substitution^c^		Patient setting	Antiviral treatment	Immunocompromised
						
		Oseltamivir	Zanamivir	Peramivir	Laninamivir	Virus isolate	Clinical specimen			
A(H1N1)pdm09; *N* = 5152	156	**151–2212**	0.1–2.7	**87–2045 (148)**	0.3–4.4 (147)	H275Y	H275Y (57) Not available (99)	Community (85 of which 44 Sapporo cluster) Hospital (17)	Yes, oseltamivir (10), oseltamivir + laninamivir (1), peramivir (11), peramivir + laninamivir (1) No (108)	Yes (2) No (87)
	12	**5.4–584**	0.7–2.0	**4.8–599 (11)**	1.0–2.7 (11)	H275Y/H mix	Not available	Community (8) Hospital (3)	Yes, oseltamivir (6), peramivir (4)	No (10)
	1	**10739**	**18.1**	**7709**	**22.0**	H275Y; I223R	H275Y; I223R	Hospital	Yes, peramivir	Unknown
	1	**16**	7.1	nd	nd	D199E	Not available	Hospital	Unknown	No
	1	**23**	4.9	4.2	3.6	I223K	I223K	Unknown	Unknown	Unknown
	2	**8.9–15**	3.1–3.2	1.8–1.7	1.7–2.0	I223T	I223T	Unknown	Unknown	Unknown
	1	**13**	7.8	5.3	2.3	I223R	Not available	Unknown	Unknown	Unknown
	1	**15**	1.2	1.3	1.2	S247G	S247G	Unknown	Unknown	Unknown
A(H3N2); *N* = 2574	1	**305**	1.4	1.4	1.1	E119V	E119V	Unknown	No	Unknown
	3	0.6 – 0.7	**16–37**	2.5–5.1	0.9–5.2	Q136K (1 mixed)	No (2) Not available (1)	Unknown	Unknown	Unknown
	1	0.9	**42**	7.6	6.3	Q136K/Q mix; D151G/D mix	Not available	Unknown	Unknown	Unknown
	1	1.2	**10**	nd	nd	T148K	Not available	Hospital	Unknown	Unknown
	1	**10**	**11**	nd	nd	N329K	Not available	Unknown	Unknown	Unknown
	1	**10**	7.8	nd	nd	S331R	Not available	Hospital	No	Unknown
	1	**10**	8.3	nd	nd	S331R; V215I	Not available	Hospital	No	Unknown
B/Victoria-lineage; *N* = 604^d^	1	1.6	2.3	**6.3**	2.5	I114R/I mix	Not available	Unknown	Unknown	Unknown
	1	**12**	**1010**	**2326**	**649**	E117G	Not available	Unknown	Unknown	Unknown
	1	3.3	0.9	**13**	0.5	H134Y/H mix	No	Community	No	Unknown
	1	1.2	1.4	**7.5**	0.8	D149N/D mix	Not available	Unknown	Unknown	Unknown
	1	1.0	**7.0**	nd	nd	N151S^d^	Not available	Hospital	No	Unknown
	1	4.7	**5.0**	nd	nd	D197N^d^	Not available	Hospital	Unknown	No
	1	1.6	1.6	**7.3**	1.2	S440L/S mix	Not sequenced	Unknown	Unknown	Unknown
	1	3.4	3.0	**5.0**	4.6	M449V	Not available	Unknown	Unknown	Unknown
	3	0.6–4.1	**0.7–8.7**	**0.9–6.5**	0.7–4.7	None	Not available (2) Not sequenced (1)	Community (2) Unknown(1)	Unknown	No (2) Unknown (1)
	1	1.0	0.9	**9.3**	0.9	I348N	Not available	Unknown	Unknown	Unknown
B/Yamagata-lineage; *N* = 2311	1	**6.8**	1.3	3.1	2.8	N151T	No	Unknown	Unknown	Unknown
	2	**4.8–6.3**	**2.8–32**	**5.5–8.2**	**3.5–9.0**	D197N	Not available	Community	Yes, zanamivir (1) Unknown (1)	No
	1	0.4	**5.6**	2.2	2.2	S249N	S249N	Community	Unknown	Unknown
	1	**7.1**	0.7	**103**	0.6	H273Y	Unknown	Unknown	Unknown	Unknown
	3	**1.7–10**	**0.9–5.3**	**2.0–7.8**	**0.6–5.1**	None	Not available	Community (2) Unknown (1)	No (1) Unknown (2)	No (2) Unknown (1)

a Between brackets the number of viruses for which data was reported if less than the number reported in column ‘*n*’. RI = reduced inhibition; HRI = highly reduced inhibition; nd = not done; None = no amino acid substitutions compared to viruses with NI phenotype.

b The values shown are ranges of fold-changes. If a range includes RI or HRI fold-change values the range is displayed underlined and in bold typeface. If a range includes only NI values, the range is displayed in plain text. Inhibition category thresholds for A viruses are: NI < 10-fold, RI 10 to 100-fold, HRI > 100-fold; and for B viruses: NI < 5-fold, RI 5 to 50-fold, HRI > 50-fold.

c Amino acid position numbering is A subtype and B type specific.

d 65 of these viruses are B/Yamagata-lineage haemagglutinin (HA) – B/Victoria-lineage neuraminidase (NA) reassortants; because the NA defines the IC_50_ values these viruses are listed in the B/Victoria-lineage category. One of these viruses had NA N151S and one NA D197N substitutions.

**Table 2:** Frequency of amino acid substitutions in NAs, submitted to GISAID and NCBI sequence databases, known to occur clinically and cause clinical resistance.^a^

Type/subtype	NA amino acid substitution^b^	No. of sequences containing the substitution (%)^c^	Home country patient (*n*)	Included in phenotypic analysis^d^
A(N1)				
	H275Y + I223R	1 (0.1%)	Japan (1)	Yes (HRI to oseltamivir and peramivir; RI to zanamivir and peramivir)
	H275Y	148 (11%)	Australia (1)	Yes (HRI to oseltamivir and peramivir)
			China (1)	Yes (HRI to oseltamivir and peramivir)
			China (1)	Yes (HRI to oseltamivir)
			China (4)	No
			Dominican Republic (1)	Yes (HRI to oseltamivir and peramivir)
			Haiti (1)	Yes (HRI to oseltamivir and peramivir)
			Japan (86)	Yes (HRI to oseltamivir and peramivir)
			Japan (1)	No
			Mexico (1)	Yes (HRI to oseltamivir and peramivir)
			New Caledonia (1)	Yes (HRI to oseltamivir and peramivir)
			Spain (1)	Yes (HRI to oseltamivir)
			United States (48)	Yes (HRI to oseltamivir and peramivir)
			United States (1)	No
	H275Y/H	16 (1%)	Brazil (1)	Yes (HRI to oseltamivir and peramivir)
			China (1)	Yes (HRI to oseltamivir)
			Japan (2)	Yes (HRI to oseltamivir and peramivir)
			Japan (2)	Yes (HRI to oseltamivir and RI to peramivir)
			Japan (2)	Yes (RI to oseltamivir and HRI to peramivir)
			Japan (2)	Yes (RI to oseltamivir and peramivir)
			Japan (1)	Yes (RI to oseltamivir)
			Japan (1)	Yes (RI to peramivir)
			Japan (1)	No
			Paraguay (1)	Yes (RI to oseltamivir)
			United States (2)	Yes (NI)

a As listed in the table on the WHO website, available at: http://www.who.int/influenza/gisrs_laboratory/antiviral_susceptibility/nai_overview/en/; accessed 15 January 2015.

b Amino acid position numbering is N1 specific.

c Percentage based on the number of sequences in the final data set after curation – see Supplementary Table 2.

d Yes indicates that the virus was analysed by a WHO CC. HRI = highly reduced inhibition, RI = reduced inhibition, NI = normal inhibition, assessed by in *vitro* assay for the neuraminidase inhibitors indicate.

**Table 3:** Frequency of amino acid substitutions in NAs, submitted to GISAID and NCBI sequence databases, known to occur clinically but currently of unknown impact, that cause reduced sensitivity *in vitro*.^a^

Type/subtype	NA amino acid substitution^b^	No. of sequences containing the substitution (%)^c^	Home country patient (*n*)	Included in phenotypic analysis^d^
A(N1)	D199N	25 (1.9 %)	Bulgaria (7)	Yes (NI)
			Dominican Republic (1)	Yes (NI)
			Georgia (1)	Yes (NI)
			Germany (1)	Yes (NI)
			Italy (1)	No
			Italy (2)	Yes (NI)
			Japan (2)	Yes (NI)
			Latvia (1)	Yes (NI)
			Malaysia (1)	Yes (NI)
			Poland (2)	No
			United Kingdom (1)	No
			United States (5)	Yes (NI)
	I223R	2 (0.2%)	Belgium (1)	No
			Malaysia (1)	Yes (RI to oseltamivir)
	N295S	0		
A(N2)	E119V	2 (0.2%)	United Kingdom (1)	Yes (NI)
			United States (1)	Yes (HRI to oseltamivir)
	R292K	0		
	N294S	0		
B^e^	R150K	0		
	D197E	0		
	D197N	4 (0.5%)	China (HA Yam/NA Vic) (1)	Yes (RI to zanamivir and oseltamivir)
			China (HA Yam/NA Yam) (1)	No
			Japan (HA Yam/NA Yam) (1)	Yes (RI to zanamivir, oseltamivir, peramivir and laninamivir)
			Japan (HA Yam/NA Yam) (1)	Yes (RI to oseltamivir and peramivir)
	I221T	0		
	N294S	0		
	G407S	0		

a As listed in the table on the WHO website, available at: http://www.who.int/influenza/gisrs_laboratory/antiviral_susceptibility/nai_overview/en/; accessed 15 January 2015.

b Amino acid position numbering is A subtype and B type specific.

c Percentage based on the number of sequences in the final data set after curation – see Supplementary Table 2.

d Yes indicates that the virus was analysed by a WHO CC. NI = normal inhibition; RI = reduced inhibition; HRI = highly reduced inhibition, assessed by in vitro assay for the neuraminidase inhibitors (NAIs) indicated.

e The H273Y substitution found in the WHO CC data was not included here, because it did not fulfil the requirements for screening: a new substitution should be present in the clinical specimen and more than a single occurrence if in a patient who has not been treated with a NAI.
