# Supplementary material for: Global update on the susceptibility of human influenza viruses to neuraminidase inhibitors, 2013–2014
Source: Antiviral Res. Author manuscript; Available in PMC 2022 Apr 25. (PMC9036627; doi:10.1016/j.antiviral.2015.02.003)
Supplement: mmc1 [file NIHMS1796922-supplement-mmc1.doc]

Supplementary Table 1.

NA inhibition assay methodology in WHO CCs.

| **WHO CCs** | **Procedure used for IC_50_ determination** | | | | **Antivirals available** |
| --- | --- | --- | --- | --- | --- |
|  | **Reagents** | **Protocol** | **Plate reader** | **Software for IC_50_ determination** |  |
| Atlanta, United States | NA-Fluor Influenza Neuraminidase Assay Kit (Applied Biosystems) | Atlanta WHO CC optimized fluorescence-based assay | Victor^3^ V (PerkinElmer)  Synergy Neo (BioTek) | JASPR v2.1 (CDC) | Oseltamivir  Zanamivir  Peramivir  Laninamivir |
| Beijing, China | NA-Fluor Influenza Neuraminidase Assay Kit (Applied Biosystems) | Beijing WHO CC optimized fluorescence-based assay | Envision (PerkinElmer) | GraphPad Prism 5 (GraphPad Software) | Oseltamivir  Zanamivir |
| London, United Kingdom | In-house (MUNANA) | London WHO CC optimized fluorescence-based assay | Infinite F200 PRO (Tecan) | Excel and GraFit 7 (Erithacus Software) | Oseltamivir  Zanamivir |
| Melbourne, Australia | In-house (MUNANA) | Melbourne WHO CC optimized fluorescence-based assay | Fluoroskan Ascent FL Microplate Fluorometer (Thermo Scientific) | JASPR v2.1 (CDC) | Oseltamivir  Zanamivir  Peramivir  Laninamivir |
| Tokyo, Japan | NA-Fluor Influenza Neuraminidase Assay Kit (Applied Biosystems) | Tokyo WHO CC optimized fluorescence-based assay | Mithras LB940 (Berthold) | MikroWin 2000 (Mikrotek Laborsysteme) | Oseltamivir  Zanamivir  Peramivir  Laninamivir |
