# Supplementary material for: Global update on the susceptibility of human influenza viruses to neuraminidase inhibitors, 2013–2014
Source: Antiviral Res. Author manuscript; Available in PMC 2022 Apr 25. (PMC9036627; doi:10.1016/j.antiviral.2015.02.003)
Supplement: mmc2 [file NIHMS1796922-supplement-mmc2.doc]

Supplementary Table 2.

Selection and curation of NA sequence data from GISAID and NCBI sequence databases for analysis ^a^.

| Dataset | Public sequence database | Number of sequences by influenza virus subtype/lineage | | | |
| --- | --- | --- | --- | --- | --- |
|  |  | A(H1N1)pdm09 | A(H3N2) | B/Yam | B/Vic |
| *No. of sequences available on-line with:* | GISAID | 1355 | 1186 | 538 | 292 |
| - Minimum length: 1407 nucleotides (H1N1pdm09 and H3N2); 1398 nucleotides (B) | NCBI-IVR | 115 | 246 | 165 ^b^ | |
| *Final dataset following removal of:* | GISAID | 1245 | 1043 | 519 | 278 |
| - Sequences without a complete coding region | |  |  |  |  |
| - Repeated sequences between and within databases ^c,d^ | NCBI-IVR | 83 | 27 | 2 ^b^ | 9 ^b^ |
| - Sequential degenerate nucleotides ^e^ |  |  |  |  |  |
| A(H3N2)v sequences ^f^ |  |  |  |  |  |
| **Total** |  | **1328** | **1070** | **521** | **287** |

^a^ Global Initiative on Sharing All Influenza Data (GISAID) at www.gisaid.org and National Center for Biotechnology Information Influenza Virus Resource (NCBI-IVR) at [www.ncbi.nlm.nih.gov/genomes/FLU/FLU.html](http://www.ncbi.nlm.nih.gov/genomes/FLU/FLU.html); accessed 15 January 2015.

^b^ In the NCBI-IVR database, lineage information is not present for most influenza B viruses. Lineage designation was performed after generating independent phylogenies based on HA and NA genes.

^c^ Sequences repeated between the two databases were downloaded from the GISAID database only.

^d^ Sequences repeated within the same database were analysed for redundancy (Jalview software); when the sequences were redundant, one of the sequences was maintained in the dataset; when the sequences were different, all sequences for that virus were removed from the dataset.

^e^ Sequences with potential degenerate nucleotides were not removed unless they contained 3 or more sequential degenerate nucleotides (with none or few nucleotides separating them).

^f^ Analysis of the GISAID sequence data for A(H3N2) revealed that A(H3N2)v sequences were included, so datasets without A(H3N2)v sequences were constructed and analysed. When removing the A(H3N2)v sequences it was noticed that other sequences from the same country displayed the same NA sequence pattern at certain nucleotide positions (76G; 90G; 118C; 123T; 127G; 150A; numbering is N2 specific). By considering the NA sequence pattern for removal of sequences, a further 12 sequences were removed to produce the dataset analysed here.
