# Supplementary material for: Global update on the susceptibility of human influenza viruses to neuraminidase inhibitors, 2013–2014
Source: Antiviral Res. Author manuscript; Available in PMC 2022 Apr 25. (PMC9036627; doi:10.1016/j.antiviral.2015.02.003)
Supplement: mmc3 [file NIHMS1796922-supplement-mmc3.doc]

**Takashita et al., Global update on the susceptibility of human influenza viruses to neuraminidase inhibitors, 2013-2014**

**Supplementary Table 3.**

List of submitting and originating laboratories of the sequences retrieved from the GISAID sequence database (Collection period, May 20, 2013 to May 18, 2014), *accessed as of August 19, 2014*.

| **Submitting laboratory** | **Originating laboratory** | **Number of sequences** |
| --- | --- | --- |
| **Atlanta WHO CC, United States** |  | **1202** |
|  | [Argentina] CEMIC University Hospital | 1 |
|  | [Argentina] Instituto Nacional de Enfermedades Infecciosas | 9 |
|  | [Australia] WHO Collaborating Centre for Reference and Research on Influenza | 2 |
|  | [Bahrain] Ministry of Health | 3 |
|  | [Bangladesh] Institute of Epidemiology Disease Control and Research (IEDCR) & Bangladesh National Influenza Centre (NIC) | 28 |
|  | [Bolivia] CENETROP | 4 |
|  | [Bolivia] Instituto Nacional de Laboratoriosde Salud (INLASA) | 1 |
|  | [Brazil] Instituto Adolfo Lutz | 18 |
|  | [Brazil] National Influenza Center | 1 |
|  | [Brazil] Oswaldo Cruz Institute - FIOCRUZ - Laboratory of Respiratory Viruses and Measles (LVRS) | 6 |
|  | [Burkina Faso] IRSS | 6 |
|  | [Canada] National Microbiology Laboratory, Health Canada | 35 |
|  | [Chile] Instituto de Salud Publica de Chile | 20 |
|  | [China] WHO Chinese National Influenza Center | 15 |
|  | [Costa Rica] Laboratorio Nacional de Influenza | 8 |
|  | [Dominican Republic] Laboratorio de Investigacion / Centro de Educacion Medica y Amistad Dominico Japones (CEMADOJA) | 6 |
|  | [Ecuador] INSPI | 1 |
|  | [El Salvador] Contiguo a Hospital Rosales | 7 |
|  | [Ethiopia] Ethiopian Health and Nutrition Research Institute (EHNRI) | 10 |
|  | [French Guiana] National Influenza Center French Guiana and French Indies | 12 |
|  | [Guatemala] Laboratorio Nacional De Salud Guatemala | 2 |
|  | [Hong Kong (SAR)] Government Virus Unit | 17 |
|  | [India] National Institute of Virology | 8 |
|  | [Indonesia] Ministry of Health, NIHRD | 8 |
|  | [Jamaica] University of the West Indies | 2 |
|  | [Kenya] CDC-Kenya | 6 |
|  | [Korea, Republic of] National Institute of Health | 5 |
|  | [Lao, People's Democratic Republic] National Center for Laboratory and Epidemiology | 6 |
|  | [Mexico] Laboratorio de Virus Respiratorio | 12 |
|  | [New Zealand] Institute of Environmental Science & Research | 5 |
|  | [Nicaragua] Laboratorio de Virologia, Direccion de Microbiologia | 3 |
|  | [Nigeria] National Influenza Reference Laboratory | 2 |
|  | [Oman] Central Public Health Laboratory, Ministry of Health | 1 |
|  | [Pakistan] National Institute of Health | 7 |
|  | [Panama] Instituto Conmemorativo Gorgas de Estudios de la Salud | 5 |
|  | [Paraguay] Central Laboratory of Public Health | 4 |
|  | [Peru] NAMRU-6 | 3 |
|  | [Peru] US NAMRU-6 | 14 |
|  | [Puerto Rico] Puerto Rico Department of Health | 11 |
|  | [Russian Federation] Ivanovsky Research Institute of Virology RAMS | 1 |
|  | [Russian Federation] Russian Academy of Medical Sciences | 8 |
|  | [Rwanda] Rwanda Biomedical Center, National Reference Laboratory | 1 |
|  | [Senegal] Institut pasteur de Dakar | 13 |
|  | [Taiwan] ADImmune Corporation | 2 |
|  | [Tanzania, United Republic of] National Influenza Lab | 2 |
|  | [Thailand] WHO National Influenza Centre, National Institute of Medical Research (NIMR) | 14 |
|  | [Trinidad and Tobago] Caribbean Epidemiology Center | 16 |
|  | [Ukraine] Institute of Epidemiology and Infectious Diseases AMS of Ukraine | 10 |
|  | [United Kingdom] National Institute for Medical Research | 3 |
|  | [United States] ADPH Bureau of Clinical Laboratories | 7 |
|  | [United States] Alaska State Public Health Laboratory | 1 |
|  | [United States] Alaska State Virology Lab | 15 |
|  | [United States] Arizona Department of Health Services | 12 |
|  | [United States] Arkansas Department of Health | 11 |
|  | [United States] Baylor College of Medicine | 2 |
|  | [United States] California Department of Health Services | 35 |
|  | [United States] Colorado Department of Health Lab | 15 |
|  | [United States] Connecticut Department. of Public Health | 12 |
|  | [United States] Dallas County Health and Human Services | 1 |
|  | [United States] DC Public Health Lab | 1 |
|  | [United States] Delaware Public Health Lab | 14 |
|  | [United States] Florida Department of Health-Jacksonville | 23 |
|  | [United States] Florida Department of Health-Tampa | 19 |
|  | [United States] Georgia Public Health Laboratory | 10 |
|  | [United States] Houston Department of Health and Human Services | 1 |
|  | [United States] Illinois Department of Public Health-Chicago | 8 |
|  | [United States] Indiana State Department of Health Laboratories | 14 |
|  | [United States] Iowa State Hygienic Laboratory | 16 |
|  | [United States] Kansas Department of Health and Environment | 9 |
|  | [United States] Kentucky Division of Laboratory Services | 12 |
|  | [United States] Los Angeles Quarantine Station, CDC Quarantine Epidemiology and Surveillance Team | 2 |
|  | [United States] Louisiana Department of Health and Hospitals | 18 |
|  | [United States] Maine Health and Environmental Testing Laboratory | 8 |
|  | [United States] Marshfield Clinic Research Foundation | 7 |
|  | [United States] Maryland Department of Health and Mental Hygiene | 24 |
|  | [United States] Massachusetts Department of Public Health | 21 |
|  | [United States] Michigan Department of Community Health | 5 |
|  | [United States] Minnesota Department of Health | 14 |
|  | [United States] Mission Hospital Microbiology Lab | 1 |
|  | [United States] Mississippi Public Health Laboratory | 11 |
|  | [United States] Missouri Department. of Health & Senior Services | 8 |
|  | [United States] Montana Laboratory Services Bureau | 9 |
|  | [United States] Montana Public Health Laboratory | 3 |
|  | [United States] Naval Health Research Center | 1 |
|  | [United States] Nebraska Public Health Lab | 12 |
|  | [United States] Nevada State Health Laboratory | 11 |
|  | [United States] New Hampshire Public Health Laboratories | 17 |
|  | [United States] New Jersey Department of Health & Senior Services | 15 |
|  | [United States] New Mexico Department of Health | 17 |
|  | [United States] New York City Department of Health | 9 |
|  | [United States] New York State Department of Health | 23 |
|  | [United States] North Carolina State Laboratory of Public Health | 28 |
|  | [United States] North Dakota Department of Health | 6 |
|  | [United States] Ohio Department of Health Laboratories | 4 |
|  | [United States] Oklahoma State Department of Health | 12 |
|  | [United States] Oregon Public Health Laboratory | 10 |
|  | [United States] Pennsylvania Department of Health | 25 |
|  | [United States] Rhode Island Department of Health | 6 |
|  | [United States] Seattle & King County Public Health Lab | 2 |
|  | [United States] South Carolina Department of Health | 9 |
|  | [United States] South Dakota Public Health Lab | 5 |
|  | [United States] Southern Nevada Public Health Lab | 14 |
|  | [United States] Spokane Regional Health District | 11 |
|  | [United States] St. Judes Childrens Research Hospital | 1 |
|  | [United States] State of Hawaii Department of Health | 27 |
|  | [United States] State of Idaho Bureau of Laboratories | 6 |
|  | [United States] Tennessee Department of Health Laboratory-Nashville | 1 |
|  | [United States] Texas Department of State Health Services-Laboratory Services | 32 |
|  | [United States] U.S. Air Force School of Aerospace Medicine | 6 |
|  | [United States] University of Michigan SPH EPID | 5 |
|  | [United States] University of Virginia, Medical Labs/Microbiology | 6 |
|  | [United States] UPMC-CLB Dept of Microbiology | 6 |
|  | [United States] Utah Department of Health | 24 |
|  | [United States] Vermont Department of Health Laboratory | 4 |
|  | [United States] Virginia Division of Consolidated Laboratories | 12 |
|  | [United States] Washington State Public Health Laboratory | 12 |
|  | [United States] West Virginia Office of Laboratory Services | 7 |
|  | [United States] Westchester Medical Center | 1 |
|  | [United States] Wisconsin State Laboratory of Hygiene | 20 |
|  | [United States] Wyoming Public Health Laboratory | 9 |
|  | [United States] Yale New Haven Hospital, Clinical Virology Laboratory | 1 |
|  | [Uruguay] Departamento de Laboratorio de Salud Publica | 6 |
|  | [Venezuela, Bolivarian Republic of] Instituto Nacional de Higiene "Rafael Rangel" | 5 |
|  | [Vietnam] National Institute of Hygiene and Epidemiology | 11 |
|  | [Vietnam] Pasteur Institute, Influenza Laboratory | 8 |
|  | Unknown | 2 |
| **Beijing WHO CC, China** |  | **83** |
|  | [China] WHO Chinese National Influenza Center | 83 |
| **London WHO CC, United Kingdom** |  | **725** |
|  | [Albania] Institute of Public Health | 11 |
|  | [Algeria] Institut Pasteur d'Algerie | 1 |
|  | [Austria] University of Vienna | 13 |
|  | [Belgium] Scientific Institute of Public Health | 19 |
|  | [Bosnia and Herzegovina] University of Sarajevo | 1 |
|  | [Bulgaria] National Centre of Infectious and | 25 |
|  | [Cameroon] Centre Pasteur du Cameroun | 28 |
|  | [China] WHO Chinese National Influenza Center | 3 |
|  | [Cote d'Ivoire] Pasteur Institut of Côte d'Ivoire | 13 |
|  | [Croatia] Croatian Institute of Public Health | 4 |
|  | [Cyprus] Nicosia General Hospital | 10 |
|  | [Denmark] Statens Serum Institute | 3 |
|  | [Egypt] VACSERA | 1 |
|  | [Finland] National Institute for Health and Welfare | 6 |
|  | [France] CRR virus Influenza region Sud | 17 |
|  | [France] Institut Pasteur | 7 |
|  | [Georgia] National Centre for Disease Control and Public Health | 12 |
|  | [Germany] Robert-Koch-Institute | 27 |
|  | [Ghana] University of Ghana | 26 |
|  | [Greece] Aristotelian University of Thessaloniki | 1 |
|  | [Greece] Hellenic Pasteur Institute | 6 |
|  | [Hong Kong (SAR)] Government Virus Unit | 16 |
|  | [Iceland] Landspitali - University Hospital | 16 |
|  | [Iran, Islamic Republic of] Tehran University of Medical Sciences | 7 |
|  | [Ireland] National Virus Reference Laboratory | 17 |
|  | [Israel] NIC | 5 |
|  | [Italy] Istituto Superiore di Sanità | 33 |
|  | [Jordan] Laboratory Directorate | 15 |
|  | [Kazakhstan] National Reference Laboratory | 13 |
|  | [Latvia] State Agency, Infectology Center of Latvia | 11 |
|  | [Macedonia, the former Yogoslav Republic of] Republic Institute for Health Protection | 3 |
|  | [Madagascar] Institut Pasteur de Madagascar | 11 |
|  | [Malta] Mater Dei Hospital | 4 |
|  | [Mauritius] Central Health Laboratory | 3 |
|  | [Moldova, Republic of] National Centre for Preventive Medicine | 2 |
|  | [Netherlands] Erasmus University of Rotterdam | 4 |
|  | [Norway] WHO National Influenza Centre | 36 |
|  | [Poland] National Institute of Public Health - National Institute of Hygiene | 18 |
|  | [Portugal] Instituto Nacional de Saude | 8 |
|  | [Romania] Cantacuzino Institute | 12 |
|  | [Russian Federation] Ivanovsky Research Institute of Virology RAMS | 5 |
|  | [Russian Federation] WHO National Influenza Centre Russian Federation | 12 |
|  | [Senegal] Institut Pasteur de Dakar | 6 |
|  | [Slovakia] National Public Health Institute of Slovakia | 9 |
|  | [Slovenia] Laboratory for Virology, National Institute of Public Health | 18 |
|  | [South Africa] Sandringham, National Institute for Communicable D | 10 |
|  | [Spain] Barcelona, Facultad de Medicina | 9 |
|  | [Spain] Instituto de Salud Carlos III | 26 |
|  | [Spain] Universidad de Valladolid | 7 |
|  | [Sweden] Swedish Institute for Infectious Disease Control | 10 |
|  | [Switzerland] Hopital Cantonal Universitaire de Geneves | 15 |
|  | [Togo] Institute National D'Hygiene | 7 |
|  | [Tunisia] Hopital Charles Nicolle | 1 |
|  | [Turkey] Istanbul University | 5 |
|  | [Turkey] Refik Saydam National Public Health Agency | 45 |
|  | [Ukraine] Institute of Epidemiology and Infectious Diseases AMS of Ukraine | 21 |
|  | [Ukraine] Ministry of Health of Ukraine | 14 |
|  | [United Kingdom] Health Protection Agency | 18 |
|  | [Zambia] University Teaching Hospital | 7 |
|  | Unknown | 12 |
| **Melbourne WHO CC, Australia** |  | **282** |
|  | [Australia] Austin Health | 8 |
|  | [Australia] Canberra Hospital | 12 |
|  | [Australia] Clinical Virology Unit, CDIM | 11 |
|  | [Australia] Institute of Medical and Veterinary Science (IMVS) | 33 |
|  | [Australia] John Hunter Hospital | 1 |
|  | [Australia] John Hunter Hospital, Virology Unit, Clinical Microbiology | 3 |
|  | [Australia] Monash Medical Centre | 27 |
|  | [Australia] Pathwest QE II Medical Centre | 10 |
|  | [Australia] Princess Margaret Hospital for Children | 4 |
|  | [Australia] Queensland Health Scientific Services | 27 |
|  | [Australia] Royal Chidrens Hospital | 4 |
|  | [Australia] Royal Darwin Hospital | 27 |
|  | [Australia] Royal Hobart Hospital | 15 |
|  | [Australia] Victorian Infectious Diseases Reference Laboratory | 2 |
|  | [Australia] Westmead Hospital | 5 |
|  | [Cambodia] Institute Pasteur du Cambodia | 10 |
|  | [Fiji] National Centre for Scientific Services for Virology and Vector Borne Diseases | 3 |
|  | [Malaysia] Institut Penyelidikan Perubatan | 3 |
|  | [New Caledonia] Institut Pasteur New Caledonia | 6 |
|  | [New Zealand] Auckland Healthcare | 1 |
|  | [New Zealand] Auckland Hospital | 2 |
|  | [New Zealand] Canterbury Health Services | 9 |
|  | [New Zealand] Institute of Environmental Science and Research | 37 |
|  | [Papua New Guinea] Papua New Guinea Institute of Medical Research | 3 |
|  | [Philippines] Research Institute of Tropical Medicine | 1 |
|  | [Singapore] National Public Health Laboratory | 5 |
|  | [South Africa] National Institute for Communicable Disease | 2 |
|  | [Sri Lanka] Medical Research Institute | 3 |
|  | [Thailand] WHO National Influenza Centre, National Institute of Medical Research (NIMR) | 8 |
| **Tokyo WHO CC, Japan** |  | **564** |
|  | [Japan] Aichi Prefectural Institute of Public Health | 12 |
|  | [Japan] Akita Research Center for Public Health and Environment | 2 |
|  | [Japan] Aomori Prefectural Institute of Public Health and Environment | 2 |
|  | [Japan] Center for Public Health and Environment, Hiroshima Prefectural Technology Research Institute | 6 |
|  | [Japan] Chiba City Institute of Health and Environment | 10 |
|  | [Japan] Chiba Prefectural Institute of Public Health | 7 |
|  | [Japan] Ehime Prefecture Institute of Public Health and Environmental Science | 7 |
|  | [Japan] Fukui Prefectural Institute of Public Health | 8 |
|  | [Japan] Fukuoka City Institute for Hygiene and the Environment | 4 |
|  | [Japan] Fukuoka Institute of Public Health and Environmental Sciences | 3 |
|  | [Japan] Fukushima Prefectural Institute of Public Health | 5 |
|  | [Japan] Gifu Municipal Institute of Public Health | 4 |
|  | [Japan] Gifu Prefectural Institute of Health and Environmental Sciences | 5 |
|  | [Japan] Gunma Prefectural Institute of Public Health and Environmental Sciences | 6 |
|  | [Japan] Hamamatsu City Health Environment Research Center | 6 |
|  | [Japan] Hiroshima City Institute of Public Health | 3 |
|  | [Japan] Hokkaido Institute of Public Health | 8 |
|  | [Japan] Hyogo Prefectural Institute of Public Health and Consumer Sciences | 7 |
|  | [Japan] Ibaraki Prefectural Institute of Public Health | 3 |
|  | [Japan] Ishikawa Prefectural Institute of Public Health and Environmental Science | 6 |
|  | [Japan] Kagoshima Prefectural Institute for Environmental Research and Public Health | 1 |
|  | [Japan] Kanagawa Prefectural Institute of Public Health | 10 |
|  | [Japan] Kawasaki City Institute of Public Health | 6 |
|  | [Japan] Kitakyusyu City Institute of Enviromental Sciences | 5 |
|  | [Japan] Kobe Institute of Health | 5 |
|  | [Japan] Kochi Public Health and Sanitation Institute | 7 |
|  | [Japan] Kumamoto City Environmental Research Center | 3 |
|  | [Japan] Kumamoto Prefectural Institute of Public Health and Environmental Science | 6 |
|  | [Japan] Kyoto City Institute of Health and Environmental Sciences | 1 |
|  | [Japan] Mie Prefecture Health and Environment Research Institute | 11 |
|  | [Japan] Miyagi Prefectural Institute of Public Health and Environment | 5 |
|  | [Japan] Miyazaki Prefectural Institute for Public Health and Environment | 1 |
|  | [Japan] Nagano City Health Center | 5 |
|  | [Japan] Nagano Environmental Conservation Research Institute | 11 |
|  | [Japan] Nara Prefectural Institute for Hygiene and Environment | 3 |
|  | [Japan] Niigata City Institute of Public Health and Environment | 7 |
|  | [Japan] Niigata Prefectural Institute of Public Health and Environmental Sciences | 6 |
|  | [Japan] Oita Prefectural Institute of Health and Environment | 5 |
|  | [Japan] Okayama Prefectural Institute for Environmental Science and Public Health | 4 |
|  | [Japan] Okinawa Prefectural Institute of Health and Environment | 2 |
|  | [Japan] Osaka City Institute of Public Health and Environmental Sciences | 9 |
|  | [Japan] Osaka Prefectural Institute of Public Health | 16 |
|  | [Japan] Research Institute for Environmental Sciences and Public Health of Iwate Prefecture | 7 |
|  | [Japan] Saga Prefectural Institute of Public Health and Pharmaceutical Research | 1 |
|  | [Japan] Sagamihara City Laboratory of Public Health | 1 |
|  | [Japan] Saitama City Institute of Health Science and Research | 3 |
|  | [Japan] Saitama Institute of Public Health | 17 |
|  | [Japan] Sakai City Institute of Public Health | 12 |
|  | [Japan] Sapporo City Institute of Public Health | 80 |
|  | [Japan] Sendai City Institute of Public Health | 7 |
|  | [Japan] Shiga Prefectural Institute of Public Health | 5 |
|  | [Japan] Shimane Prefectural Institute of Public Health and Environmental Science | 9 |
|  | [Japan] Shizuoka City Institute of Environmental Sciences and Public Health | 5 |
|  | [Japan] Shizuoka Institute of Environment and Hygiene | 8 |
|  | [Japan] Tochigi Prefectural Institute of Public Health and Environmental Science | 7 |
|  | [Japan] Tokushima Prefectural Centre for Public Health and Environmental Sciences | 3 |
|  | [Japan] Tokyo Metropolitan Institute of Public Health | 27 |
|  | [Japan] Tottori Prefectural Institute of Public Health and Environmental Science | 3 |
|  | [Japan] Toyama Institute of Health | 3 |
|  | [Japan] Virus Research Center, Sendai Medical Center | 1 |
|  | [Japan] Wakayama City Institute of Public Health | 9 |
|  | [Japan] Wakayama Prefectural Research Center of Environment and Public Health | 9 |
|  | [Japan] Yamagata Prefectural Institute of Public Health | 11 |
|  | [Japan] Yamaguchi Prefectural Institute of Public Health and Environment | 8 |
|  | [Japan] Yokohama City Institute of Health | 22 |
|  | [Japan] Yokosuka Institute of Public Health | 2 |
|  | [Lao, People's Democratic Republic] Ministry of Health | 18 |
|  | [Mongolia] National Center for Communicable Diseases | 4 |
|  | [Nepal] National Public Health Laboratory | 4 |
|  | [Taiwan] Center for Disease Control | 34 |
|  | [Vietnam] Pasteur Institute, Influenza Laboratory | 1 |
| **Aichi Prefectural Institute of Public Health, Japan** |  | **6** |
|  | [Japan] Aichi Prefectural Institute of Public Health | 6 |
| **Cantacuzino Institute, Romania** |  | **5** |
|  | [Romania] Cantacuzino Institute | 1 |
|  | Unknown | 4 |
| **Erasmus Medical Center, Netherlands** |  | **1** |
|  | [Netherlands] Erasmus Medical Center | 1 |
| **Hangzhou Center for Disease Control and Prevention, China** |  | **2** |
|  | [China] Hangzhou Center for Disease Control and Prevention | 2 |
| **Health Protection Agency, United Kingdom** |  | **3** |
|  | [United Kingdom] Centre for Infections, Health Protection Agency | 3 |
| **Hellenic Pasteur Institute, Greece** |  | **5** |
|  | [Greece] Hellenic Pasteur Institute | 5 |
| **Hospital Clinic de Barcelona. Spain** |  | **10** |
|  | [Spain] Hospital Clinic | 9 |
|  | [Spain] Subdireccion General de Epidemiologia y Vigilancia de la Salud | 1 |
| **INSA National Institute of Health Portugal, Portugal** |  | **2** |
|  | [Portugal] Instituto Nacional de Saude | 2 |
| **Institut Pasteur, France** |  | **53** |
|  | [France] Institut Pasteur | 53 |
| **Instituto de Salud Carlos III, Spain** |  | **7** |
|  | [Spain] Instituto de Salud Carlos III | 5 |
|  | [Spain] Servicio de Microbiología Hospital San Pedro de Alcántara | 2 |
| **Istituto Superiore di Sanità, Italy** |  | **2** |
|  | [Italy] Università Cattolica del Sacro Cuore | 1 |
|  | [Italy] University of Florence | 1 |
| **National Centre for Disease Control (NCDC), Mongolia** |  | **4** |
|  | [Mongolia] National Center for Communicable Diseases | 4 |
| **National Institute for Health and Welfare, Finland** |  | **12** |
|  | [Finland] National Institute for Health and Welfare | 12 |
| **Niigata University, Japan** |  | **2** |
|  | [Japan] Niigata University | 2 |
| **Norwegian Institute of Public Health, Norway** |  | **20** |
|  | [Norway] Akershus University Hospital | 3 |
|  | [Norway] Innlandet Hospital Trust, Division Lillehammer, Department for Microbiology | 1 |
|  | [Norway] Mikrobiologisk laboratorium, Sykehuset i Vestfold | 3 |
|  | [Norway] Molde Hospital, Laboratory for Medical Microbiology | 3 |
|  | [Norway] Oslo University Hospital, Ulleval Hospital, Dept. of Microbiology | 5 |
|  | [Norway] Ostfold Hospital - Fredrikstad, Dept. of Microbiology | 1 |
|  | [Norway] St. Olavs Hospital HF, Dept. of Medical Microbiology | 2 |
|  | [Norway] Stavanger Universitetssykehus, Avd. for Medisinsk Mikrobiologi | 2 |
| **Shanghai Municipal Center for Disease Control and Prevention, China** |  | **9** |
|  | [China] Shanghai Municipal Center for Disease Control and Prevention | 9 |
| **Swedish Institute for Infectious Disease Control, Sweden** |  | **83** |
|  | Unknown | 83 |
| **WHO National Influenza Centre Russian Federation, Russian Federation** |  | **3** |
|  | [Russian Federation] WHO National Influenza Centre Russian Federation | 3 |
| **Grand Total** |  | **3085** |
